# Evaluation of diagnostic accuracy of cone beam computed tomography and multi-detector computed tomography for detection of anatomical variations in rhinoplasty

**DOI:** 10.1186/s13005-023-00401-1

**Published:** 2024-01-03

**Authors:** Abdolreza Rouientan, Mohammad Bashir Khodaparast, Yaser Safi

**Affiliations:** 1 Department of Plastic Surgery, 15 Khordad Educational Hospital, School of Medicine, Shahid Beheshti University of Medical Sciences, Tehran, Iran; 2 Department of Oral and Maxillofacial Radiology, School of Dentistry, Shahid Beheshti University of Medical Sciences, Tehran, Iran

**Keywords:** Rhinoplasty, Multi-detector computed tomography (MDCT), Cone beam computed tomography (CBCT)

## Abstract

**Background:**

Different imaging techniques, such as multi-detector computed tomography (MDCT) scan and cone beam computed tomography(CBCT), are used to check the structure of the nose before rhinoplasty. This study aimed to evaluate the accuracy of two imaging techniques, MDCT scan, and CBCT, in diagnosing structural Variations in rhinoplasty for the first time.

**Methods:**

This diagnostic accuracy study was conducted on 64 rhinoplasty candidates who complained of snoring and sleep apnea or had a positive result in the examination with Cottle’s maneuver or modified Cottle technique between February 2021 and October 2022 at 15- Khordad Hospital affiliated to Beheshti University of Medical Sciences. Before rhinoplasty, patients were randomly assigned to one of the CT and CBCT techniques with an equal ratio. Scans were assessed for the presence of Nasal septum deviation (NSD), Mucocele, Concha bullosa, and nasal septal spur by two independent radiologists. The findings of the two methods were evaluated with the results during rhinoplasty as the gold standard.

**Results:**

NSD was the most common anatomical variation based on both imaging techniques. The accuracy of CBCT for diagnosing Nasal Septum Deviation and Mucocele was 80% and 75%, respectively. The sensitivity, specificity, and accuracy of CBCT in detecting Concha bullosa were 81.3% and 83.3%, respectively. The Kappa coefficient between CBCT and intraoperative findings for diagnosing NSD and Concha Bullosa was 0.76 and 0.73, respectively (p < 0.05).

**Conclusion:**

CBCT can be considered a suitable method with high accuracy and quality to evaluate the anatomical variations before rhinoplasty.

**Supplementary Information:**

The online version contains supplementary material available at 10.1186/s13005-023-00401-1.

## Background

Rhinoplasty is one of the world’s most common and challenging cosmetic surgeries [1, 2]. Like any other surgery, rhinoplasty may be associated with complications, dissatisfaction, and even revision [3–6]. In previous studies, The rate of complications after rhinoplasty has been reported in the range of 8 to 15% [6–9]. Complications of rhinoplasty can be bleeding, infectious, dysfunction of nasal functions, aesthetic subunits, nasal obstruction, deformity, and deviation of the nose [6, 10, 11].

Various factors can affect the success rate as well as the rate of complications after rhinoplasty. Different methods are used to check the function and structure of the nose before rhinoplasty, such as rhinomanometry, anterior rhinoscopy, rhinoscopy, nasal endoscopy, Magnetic resonance imaging (MRI), and Computed Tomography (CT) Scan [12, 13]. Studies have shown that various radiography methods, including CT scans and MRI, have improved the success rate and reduced side effects after surgery [14, 15]. Conventional imaging, such as bilateral nasal plain radiography, can show the anatomy of the nasal bone and the presence of fractures; however, this technique is limited due to its two-dimensional nature [16]. CT scanning of the nasal cavity is considered a non-invasive method that provides the surgeon with appropriate information about the structure of the nose, the anterior nasal cavities, and the condition of the septums. However, there are challenges regarding the routine use of this modality before rhinoplasty [17, 18].

Multi-detector Computed Tomography (MDCT) is the primary imaging modality for accurate nasal assessments in any plane [19, 20]. Recent studies have shown that the use of cone beam computed tomography) CBCT (as one of the new methods has been of high accuracy in evaluating the structure of the nasal cavity, which can provide accurate and useful information about the bony structure of the nasal cavity and paranasal sinuses to the surgeon before rhinoplasty [17, 21]. Many studies have shown that CBTC has a higher accuracy, lower radiation dose, and lower cost in diagnosing nasal abnormalities before rhinoplasty than other radiographic methods [19, 20, 22].

Since there are many modalities for examining the structure of the nasal cavity and paranasal sinuses before rhinoplasty, each of which has its special advantages and disadvantages, examining the findings of each of these methods and comparing them with the findings during surgery can help to choose the right method of radiological evaluation before rhinoplasty. According to our knowledge, no study has investigated the diagnostic accuracy of CBCT before rhinoplasty. This study aimed to evaluate the diagnostic accuracy of CBCT and MDCT in detecting nasal and paranasal sinus anatomy and pathologies compared to clinical findings under surgery as the gold standard.

## Methods and materials

This study was approved by the research committee of Shahid Beheshti University of Medical Sciences, Tehran, Iran, with the ethical code IR.SBMU.RIDS.REC.14,001,133.

In this diagnostic study, 91 candidates for rhinoplasty with complaints of passive dyspnea, snoring, and sleep apnea or positive test results of Cottle’s maneuver and modified Cottle technique who underwent rhinoplasty between February 2021 and October 2022 at 15- Khordad Hospital affiliated to Shahid Beheshti University of Medical Sciences, were investigated. Sixty-eight consecutive patients were included in the study.

The sampling of patients was done with an accessible and easy method. Before rhinoplasty, patients were randomly evaluated with one of two imaging methods: CBCT or MDCT. Allocation of patients to imaging methods was done in a simple, random manner. The randomization process was done using Excel software and the RANDBETWEEN function. Rhinoplasty was routinely performed on the patients, and the researcher did not perform any intervention. Informed consent was obtained from included participants.

Inclusion Criteria included Candidates for rhinoplasty, positive Cottle’s maneuver and modified Cottle tests, passive dyspnea, snoring, and sleep apnea, and informed consent. Age ≥ 50 years, Pregnancy, Body Mass Index (BMI) ≥ 35, previous or present history of heart and pulmonary diseases, active infectious (viral or bacterial) rhinitis, present signs for allergic, vasomotor, atrophic, and hypertrophic rhinitis, history of previous rhinoplasty were defined as exclusion criteria.

### Imaging techniques

#### CBCT

CBCT scans were taken with HDX WILL (Dentri, Korea), with a maximum Kvp of 100 and a field of view of 18 × 16.5 cm. Images were evaluated using an On-demand 3D application (Cybermed, Seoul, Korea), version No 1.0.10, in a standardized position for paranasal assessments.

#### MDCT

MDCT scans were taken with Philips Brilliance 64 CT Scanner 64 slice, 120 kVp, paranasal sinus FOV. For each scan, whether two independent radiologists evaluated CBCT or MDCT, serial coronal sections.

Two independent radiologists evaluated all radiographic findings for both procedures. Intra-class correlation coefficients (ICC) were used to assess the reliability. The ICC for detecting radiographic findings was high (94%) in the 89–99% range.

**Fig. 1:**
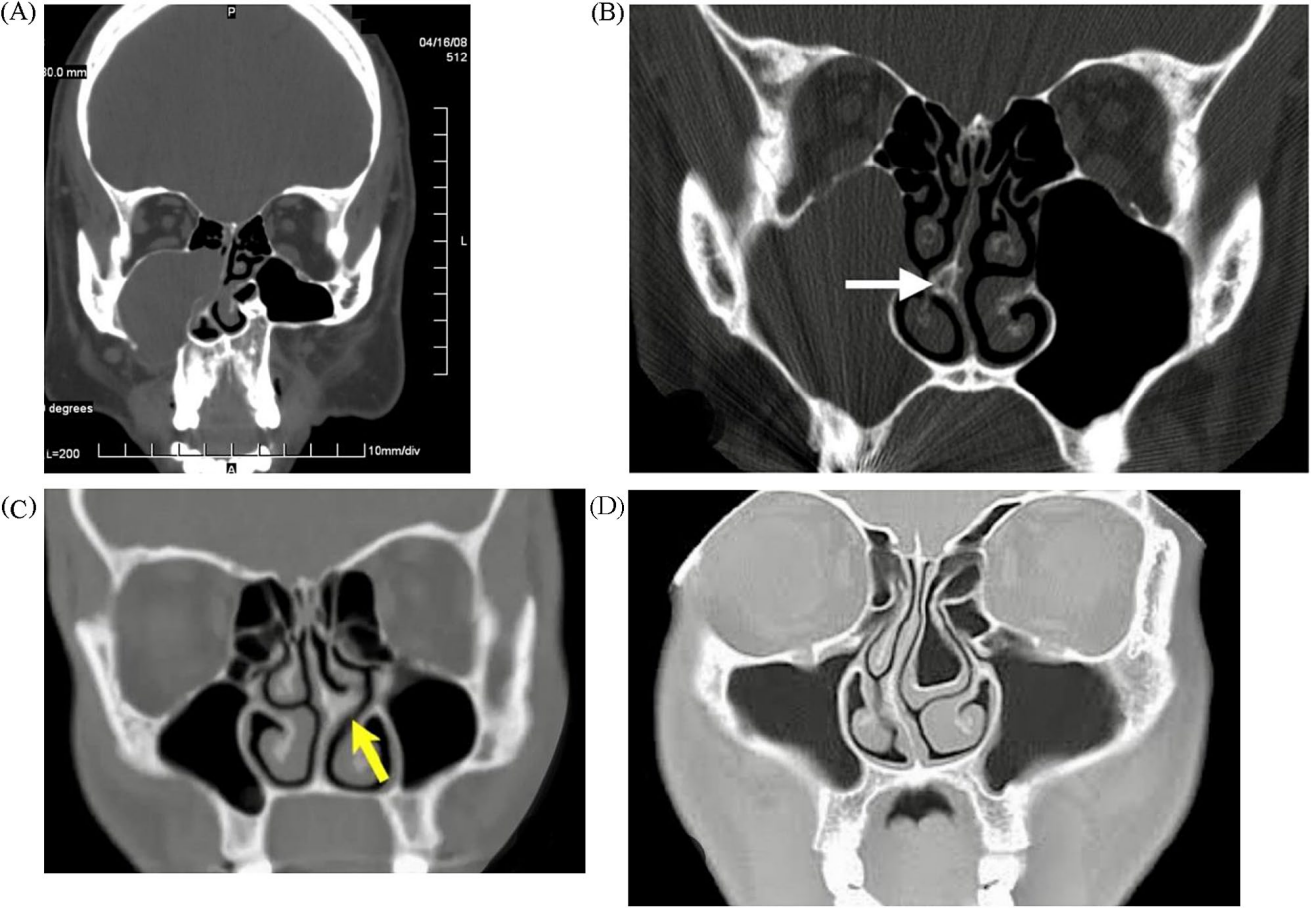
Coronal MDCT scan images. (**A**) Expansile Mucocele in the right maxillary sinus, (**B**) Nasal spur (white arrow), (**C**) Nasal septum deviation (yellow arrow), and (**D**) Concha Bullosa in the left middle turbinate

On the selected cross-sectional images, nasal and paranasal regions, detection of the following criteria was evaluated: 1-Mucocele, 2-Nasal septum deviation [23], 3-Concha bullosa and 4-Nasal septal spur (Fig. 1).

### Data collection

All information was collected by the researcher using a checklist. Demographic characteristics (age, gender, education level), radiographic findings before rhinoplasty (MDCT and CBCT), and findings during rhinoplasty were collected for all patients. Nasal Septum Deviation (NSD) detection accuracy, Mucocele detection accuracy, Concha bullosa detection accuracy, and nasal septal spur detection accuracy in two imaging methods were compared with intraoperative findings as standard criteria. All findings during surgery were evaluated and recorded by a plastic surgeon.

The diagnostic accuracy of CBCT and MDCT compared to intraoperative findings was assessed with sensitivity and specificity indices.

### Sample size collection

The appropriate sample size to carry out this study with an effect size estimate of 0.82 for the diagnostic accuracy of CBCT in the diagnosis of cleft palate criteria in infants undergoing rhinoplasty, based on the study by J Miyamoto et al. [24], with an alpha error of 5% and a 95%confidence interval using G power version 3.1 software, 33 patients were evaluated by the epidemiologist for each imaging method.

### Statistical analysis

All data were entered into a database system and evaluated using SPSS version 22(SPSS Inc., Chicago, IL, USA, 2012). Descriptive statistics were used to report qualitative variables. Quantitative variables were reported using mean and standard deviation. A chi-square statistical test was used to analyze qualitative variables in two groups. Receiver Operating Characteristic (ROC) Curve Analysis was used to evaluate the diagnostic accuracy of imaging methods in the 95% confidence interval (95% CI) to detect nasal abnormalities before rhinoplasty. In addition, Cohen’s kappa coefficient test was used to evaluate the level of agreement between different imaging methods and intraoperative findings. Classification of kappa values included “poor " (0.00), “slight " (0 to 0.20), “fair agreement” (0.21 to 0.40), “moderate agreement” (0.41 to 0.60), “substantial " (0.61 to 0.80) and " complete agreement” (> 0.8) [25]. A p of less than 0.5 was considered a statistical significance level.

## Results

### Demographic characteristics

**Fig. 2:**
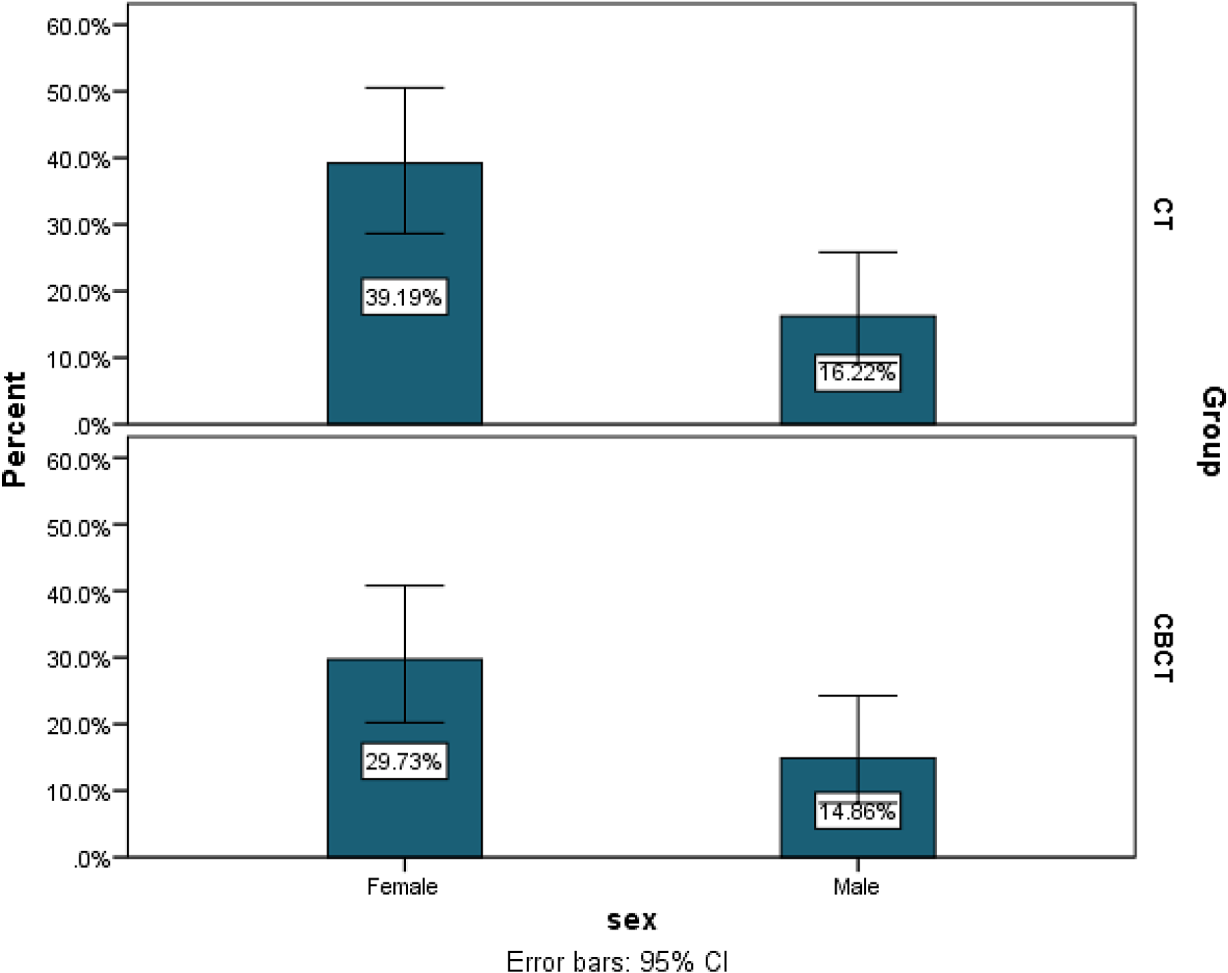
Sex distribution within the two imaging groups

Sixty-eight patients (34 patients in each group) completed the study. Of the patients, 51 (68.9%) were female, and 23 (31.1%) were female. The mean age was 29.11 ± 6.18, ranging from 15 to 49 years old. 19 (25.7%) of participants had a history of smoking. No significant difference was observed for age and gender in CBCT and MDCT groups Fig. 2.

Based on intraoperative findings, the frequency of nasal septum deviation, concha bullosa, Mucocele, and nasal septal spur in the CBCT group were 30(88.2%), 14(41.2%), 16(47.1%), and 12(35.3%), respectively. Based on intraoperative findings, no significant difference was observed in the frequency of abnormalities in the two groups (p > 0.05). The Concha bullosa and nasal septal spur were significantly better detected on CBCT than MDCT (p < 0.05). Although the frequency of Nasal SND and Mucocele detection in CBCT findings was slightly higher than in MDCT, this difference was not statistically significant (p > 0.05) (Table 1).

**Table 1:** Distribution of CBCT and MDCT findings

Variable	Imaging Methods		P value
	CBCT(N:34)	MDCT (N:34)	
Nasal Septum Deviation (n %)	28(82.4%)	26(76.5%)	0.45
Mucocele (n %)	11(32.4%)	10(29.4%)	0.51
Concha bullosa (n %)	23(67.6%)	16(47.1%)	0.015
Nasal septal spur (n %)	13(38.2%)	9(26.5%)	0.023

### Diagnostic accuracy of CBCT and MDCT for nasal septum deviation and mucocele

**Fig. 3:**
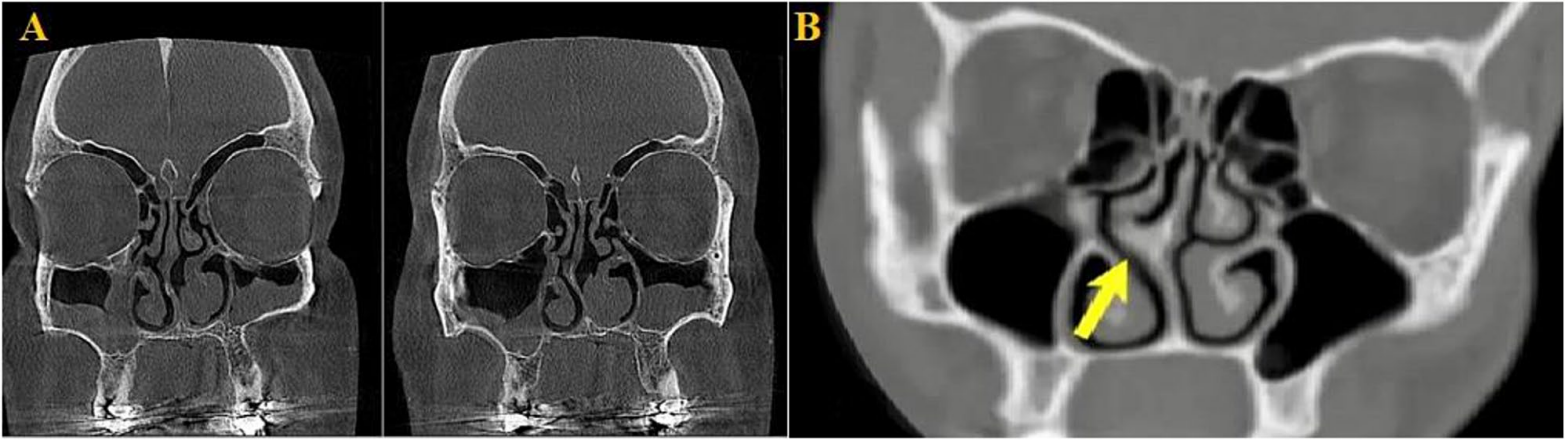
(**A**): NSD in CBCT; (**B**): NSD in MDCT

According to intraoperative clinical findings, the total prevalence of NSD was 30(88.2%) in both CBCT and MDCT groups (Fig. 3). The sensitivity and specificity of CBCT to the detection of NSD were 93.3% and 75%, respectively. The sensitivity and specificity of MDCT to detect NSD were 80% and 50%, respectively, compared to intraoperative findings. Kappa values of 0.76 and 0.22 were estimated as the level of agreement between CBCT and intraoperative findings (p: 0.001) and MDCT and intraoperative findings (p: 0.19) for diagnosing NSD, respectively.

**Fig. 4:**
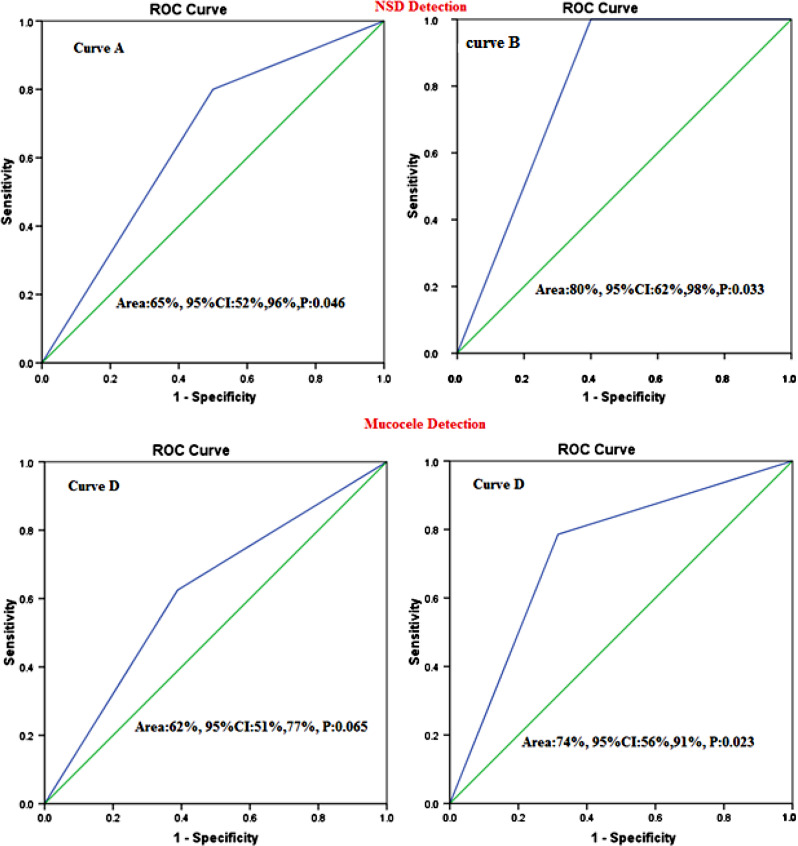
Roc curve analysis finding for the accuracy of CBCT and MDCT in diagnosing NSD and Mucocele before rhinoplasty. (Curve A: Diagnostic accuracy of MDCT for NSD, Curve B: Diagnostic accuracy of CBCT for NSD, Curve C: Diagnostic accuracy of MDCT for Mucocele, Curve D: Diagnostic accuracy of CBCT for Mucocele)

Intraoperative findings showed that mucocele frequency was 14(41.2%) and 16(47.1%) in CBCT and MDCT patients. The sensitivity and specificity of CBCT in detecting Mucocele were 78.6% and 70%, respectively (Supplement 1; Fig. 2). The sensitivity and specificity of MDCT for its diagnosis were 62.5% and 61.1% (Table 2). The accuracy of CBCT to detect NSD and Mucocele was 80% and 75%, respectively, which was statistically significant (Fig. 4- Curve B and D). The Kappa coefficient of agreement between CBCT and MDCT with intraoperative findings for Mucocele diagnosis was 0.71 and 0.34, respectively.

**Table 2:** Sensitivity, specificity, positive predictive value (PPV), negative predictive value (NPV), and kappa coefficient for nasal septum deviation and Mucocele in CBCT and MDCT compared to the gold standard

Test	Sensitivity	Specificity	PPV	NPV	Kappa coefficient (p-value)
**Nasal Septum deviation**					
CBCT Vs. Gold standard	93.3%	75%	96.6%	60%	0.76(0.001)
MDCT Vs. Gold standard	80%	50%	92.3%	75%	0.22(0.19)
**Mucocele**					
CBCT Vs. Gold standard	78.6%	70%	78.6%	82.4%	0.71(0.002)
MDCT Vs. Gold standard	62.5%	61.1%	58.5%	64.7%	0.34(0.22)

### Diagnostic accuracy of CBCT and MDCT for concha bullosa and nasal septal spur

According to intraoperative clinical findings, the total frequency of Concha bullosa was 16(47.1%) and 14(41.2%), respectively, in CBCT and MDCT group patients (Supplement 1; Fig. 3). The sensitivity and specificity of CBCT to detect Concha bullosa was 81.3% and 83.3%, respectively. The sensitivity and specificity of MDCT to the detection of Concha bullosa were 64.2% and 70%, respectively, compared to intraoperative findings (Supplement 1; Fig. 4). The Kappa coefficient between CBCT and CT with intraoperative findings for Concha Bullosa diagnosis was obtained as 0.73 and 0.40, respectively (p < 0.05) (Table 3).

**Table 3:** Sensitivity, specificity, PPV, NPV, and kappa coefficient for Concha bullosa and nasal septal spur in CBCT and MDCT compared to the gold standard

Test	Sensitivity	Specificity	PPV	NPV	Accuracy
**Concha bullosa**					
CBCT Vs. Gold standard	81.3%	83.3%	81.3%	83.3%	0.73(0.001)
MDCT Vs. Gold standard	64.2%	70%	60%	73.7%	0.40(0.02)
**Nasal septal spur**					
CBCT Vs. Gold standard	83.3.3%	81.8%	71.4%	90%	0.48(0.031)
MDCT Vs. Gold standard	64.3%	73.7%	60%	73.7%	0.21(0.3)

**Fig. 5:**
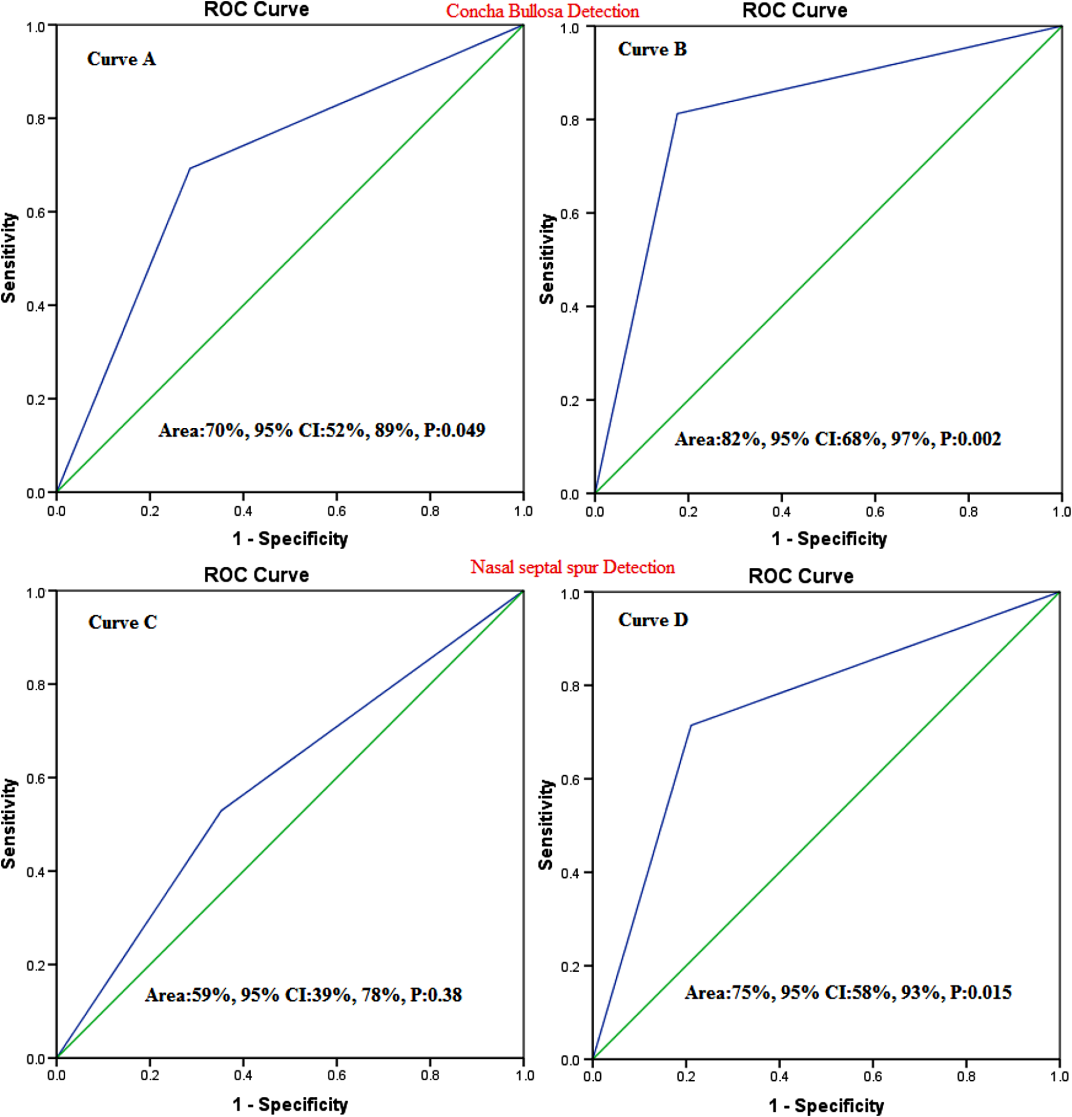
Roc curve analysis finding for the accuracy of CBCT and MDCT in diagnosing Concha bullosa and Nasal septal spur before rhinoplasty. (Curve A: Diagnostic accuracy of MDCT for Concha bullosa, Curve B: Diagnostic accuracy of CBCT for Concha bullosa, Curve C: Diagnostic accuracy of MDCT for Nasal septal spur, Curve D: Diagnostic accuracy of CBCT for Nasal septal spur)

Based on intraoperative findings, the nasal septal spur frequency in CBCT and MDCT groups was 16(47.1%) and 14(41.2%) (Supplement 1; Fig. 5). The accuracy of CBCT and MDCT in concha bullosa diagnosis was 82% and 70%, respectively. In addition, the accuracy of CBCT for diagnosing nasal septal spur was 75% and significantly better than that of MDCT (Fig. 5).

## Discussion

Patient satisfaction after rhinoplasty is one of the most important success parameters in rhinoplasty surgeries. This satisfaction depends on various factors. Studies have shown that the main reason for revision is primarily the asymmetry of the tip of the nose, followed by respiratory problems and nasal obstruction [12, 26]. Therefore, a clear image before rhinoplasty can significantly help to improve success and patient satisfaction, thereby reducing complications after surgery [27–31]. To our knowledge, no study has evaluated the accuracy of CBCT for rhinoplasty. Therefore, considering the importance of this issue, this study aimed to evaluate the accuracy of imaging findings in MDCT and CBCT methods before rhinoplasty with the findings during surgery. Our study showed that based on intraoperative findings, deviation of the nasal septum with a prevalence of 88% was the most common disorder in patients before rhinoplasty. The prevalence of concha bullosa, mucocele, and nasal septal spurs was 35 to 50%. CBCT had high sensitivity, specificity, and accuracy for diagnosing NSD. The sensitivity, specificity, and accuracy for CBCT to diagnose NSD were 93.3%, 75%, and 82%, respectively, and for MDCT, 80%, 50%, and 70%, respectively. The accuracy of CBCT for diagnosing nasal septum deviation, concha bullosa mucocele, and nasal septal spur was above 75% and in the range of 75 to 83% and was acceptable. MDCT diagnostic accuracy for nasal septum deviation, concha bullosa, Mucocele, and nasal septal spur was 65%, 62%, 70%, and 59%, respectively. Roc curve analysis showed that although the accuracy and sensitivity of CBCT were higher than MDCT in the diagnosis of NSD and Concha Bullosa, however, both CBCT and MDCT imaging techniques can help diagnose NSD and MDCT before rhinoplasty. In addition, based on the results of the ROC curve analysis, CBCT had high sensitivity and accuracy for diagnosing Mucocele and nasal septal spur. Meanwhile, MDCT did not have significant and acceptable accuracy for mucocele and nasal septal spur diagnosis. The accuracy of concha bullosa and nasal septal spur in CBCT findings was significantly higher than in MDCT.

A very limited number of studies have been conducted in this field. In a study in 2018, H Avsever et al. [32] evaluated CBCT findings from the perspective of diagnostic accuracy for paranasal sinus and NSD changes in 691 patients, showing that concha bullosa and NSD were the most common CBCT findings. In addition, they showed that CBTC could be helpful in the diagnosis of concha bullosa and septal deviation before aesthetic surgery, which was consistent with the results of our study. In another study, H Jahandideh et al. [29] in 2020, by evaluating CT and endoscopic findings before rhinoplasty in 74 patients, showed that the frequency of concha bullosa based on CT findings was 47%. Also, the frequency of NSD was 85%, which was consistent with the results of our study that the frequency of detecting deviations from the septum and concha bullosa based on CT findings was 76% and 47%, respectively.

In line with the results of our study, M Han et al. [22] in 2022, by examining CBCT findings on 60 patients, showed that, due to high accuracy, low radiation dose, and low cost, CBCT can be a useful method for initial evaluation and follow-up of patients who are candidates for cosmetic surgery. In their study, they also evaluated radiation dose and cost, while in our study, due to the study’s purpose, we could not estimate radiation dose and cost.

The importance of changing the paradigm from a 2D approach to a 3D approach in image reconstruction and the value of surface contour enhancement was shown. Based on the results of this study, CBCT had many practical applications that were very related to rhinoplasty. Surface image: Enhanced aesthetic analysis and detailed visualization of functional and bony anatomy facilitated precise surgical planning [33]. As a precise radiographic method, CBCT makes rhinoplasty procedures more predictable and efficient. The availability of spatial views, fine detail, and the possibility of easy and accurate measurement offers great potential diagnostic information. This study showed that CBCT is a user-friendly and fast technique with many advantages in planning nasal surgery [33]. It does not cause any inconvenience to the patient, has very few disadvantages, and has limited costs. A Zamani Naser et al. [34] examined the use of CBCT in measuring Iranians’ nasal bone thickness in 74 patients. They showed that nasal pyramid evaluation by CBCT can give a good view to the surgeon to perform optimal reconstructive surgery & augmentation.

In rhinoplasty surgeries, the radiation dose in radiography with CBCT is much lower than in CT scans. It prepares a much clearer picture for surgeons before surgery. Regarding patient position, imaging in this method is much easier for patients. However, the cost of CBCT is more than a CT scan.

### Study limitations

Our study had strengths and weaknesses that should be mentioned. This study’s most important weakness was the need to evaluate all patients with both CBCT and MDCT imaging techniques. In this study, we could not perform both imaging techniques on patients due to the study’s design, as well as to avoid imposing additional costs and complying with ethical principles, which can affect the results of the study to some extent. However, we partially controlled the effect of this weakness by randomly assigning study participants to one of the two imaging techniques. Assessing the diagnostic accuracy of CBCT with a precise gold standard on a suitable sample size of patients before rhinoplasty, for the first time, was the most important strength of our study.

## Conclusion

Our study showed that CBCT was highly accurate and suitable for diagnosing concha bullosa, nasal septal spur, nasal septum deviation, and Mucocele before rhinoplasty. CBCT prepares a much clearer picture for surgeons before surgery. CBCT can be considered a suitable method with high accuracy and quality to evaluate Anatomical Variations before rhinoplasty.

### Electronic supplementary material

Below is the link to the electronic supplementary material.

**Supplementary Material 1:** CT and CBCT findings (Figures 1 to 5)

## Data Availability

The datasets used and/or analysed during the current study are available from the corresponding author upon reasonable request.
