# Supplementary material for: Evaluation of diagnostic accuracy of cone beam computed tomography and multi-detector computed tomography for detection of anatomical variations in rhinoplasty
Source: Head Face Med. 2024 Jan 3;20:1. doi: 10.1186/s13005-023-00401-1 (PMC10763271; doi:10.1186/s13005-023-00401-1)
Supplement: Supplementary file 1 — Supplementary Material 1: CT and CBCT findings (Figures 1 to 5) [file 13005_2023_401_MOESM1_ESM.pptx]

## Slide 1
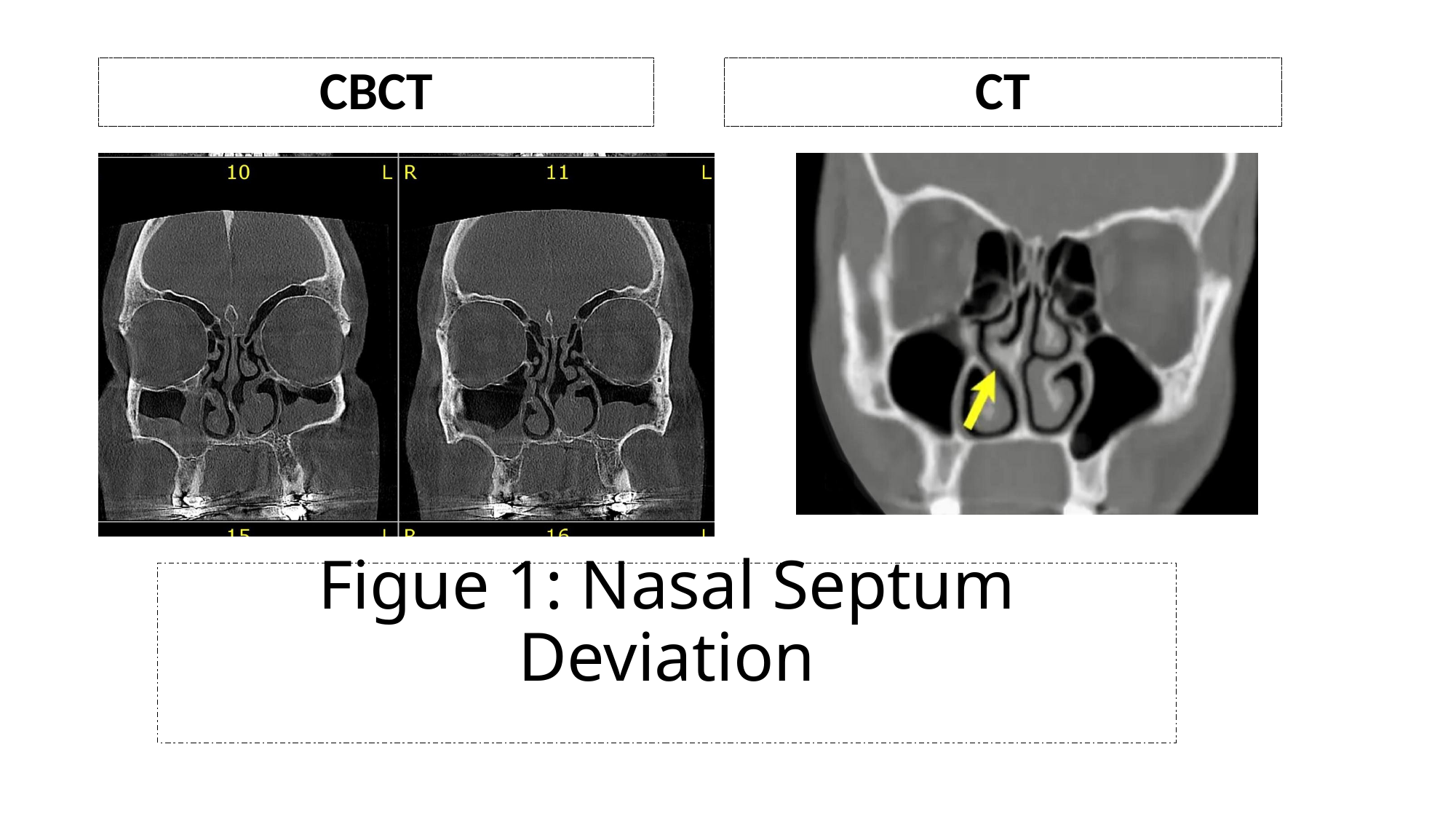

CBCT
CT
# Figue 1: Nasal Septum Deviation

## Slide 2
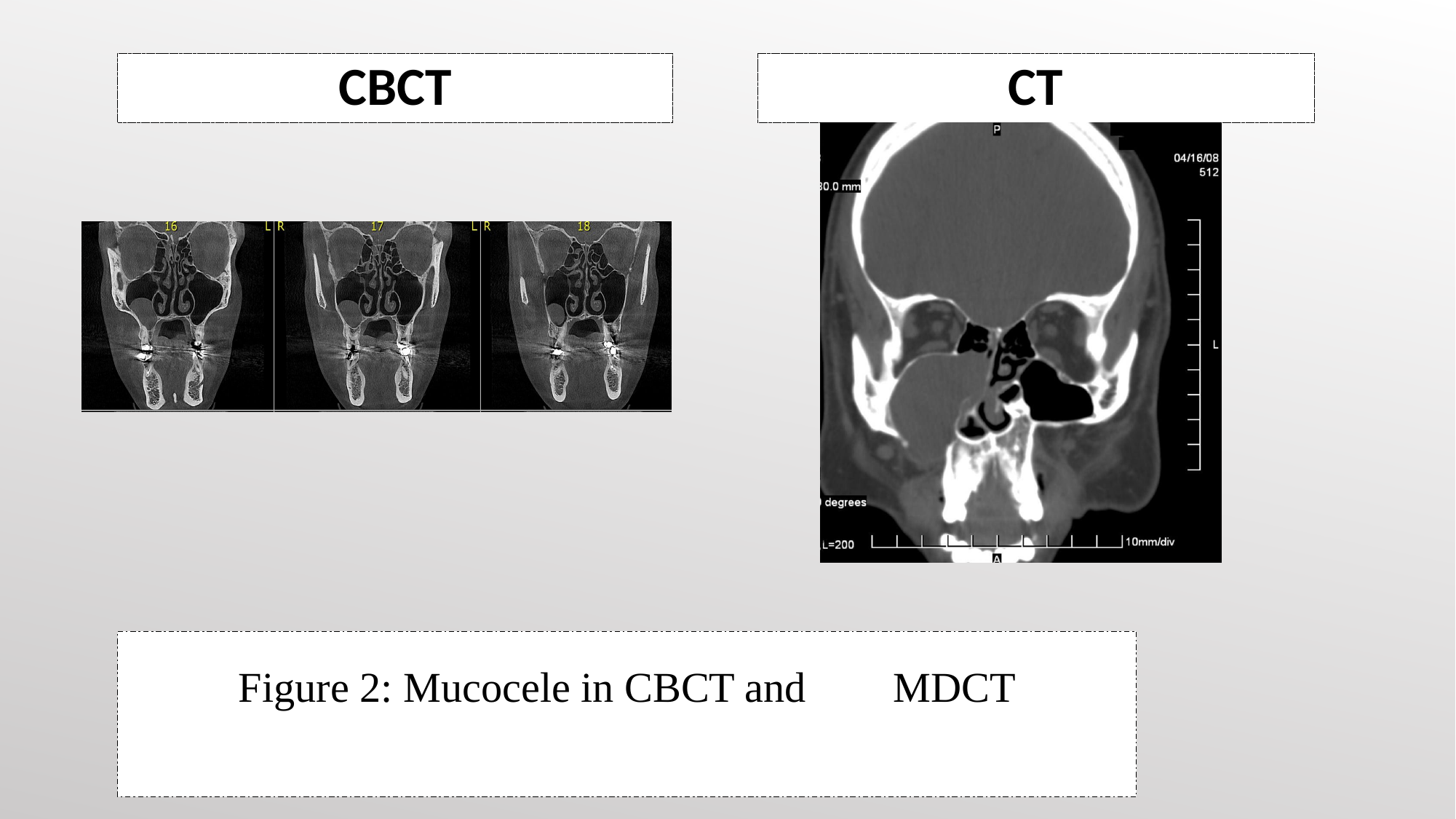

CBCT
CT
# Figure 2: Mucocele in CBCT and 	MDCT

## Slide 3
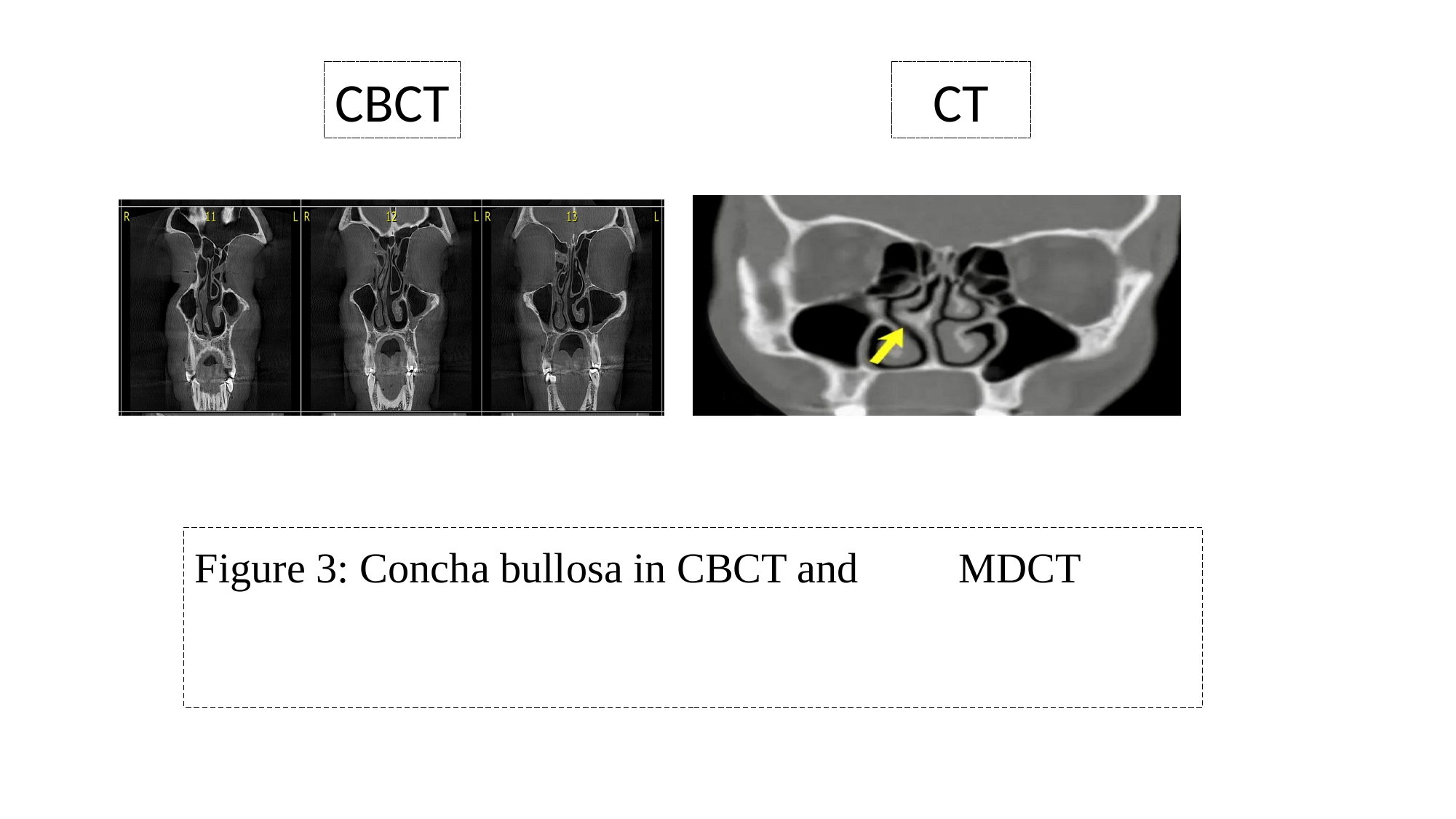

CT
CBCT
# Figure 3: Concha bullosa in CBCT and 	MDCT

## Slide 4
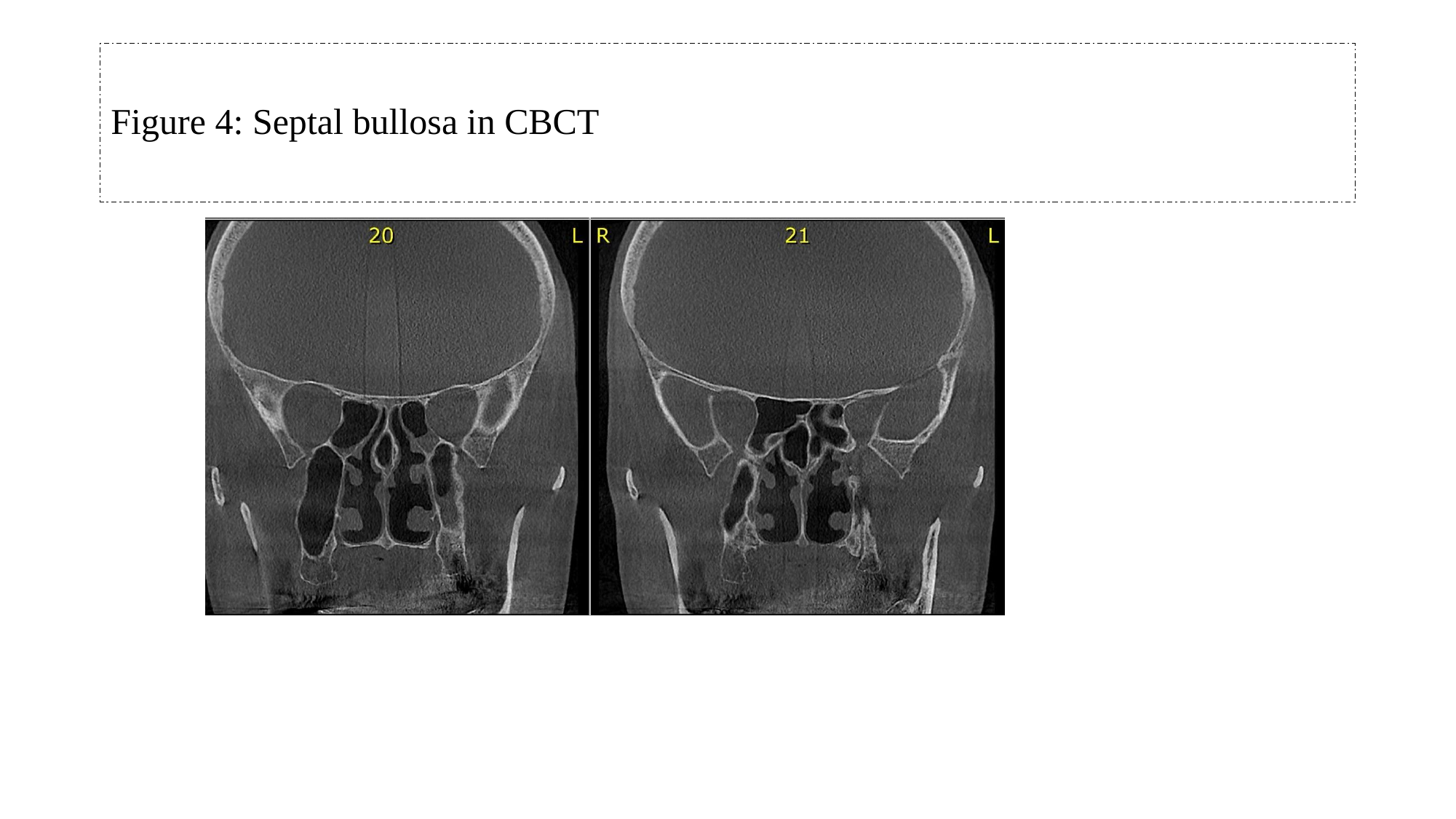

# Figure 4: Septal bullosa in CBCT

## Slide 5
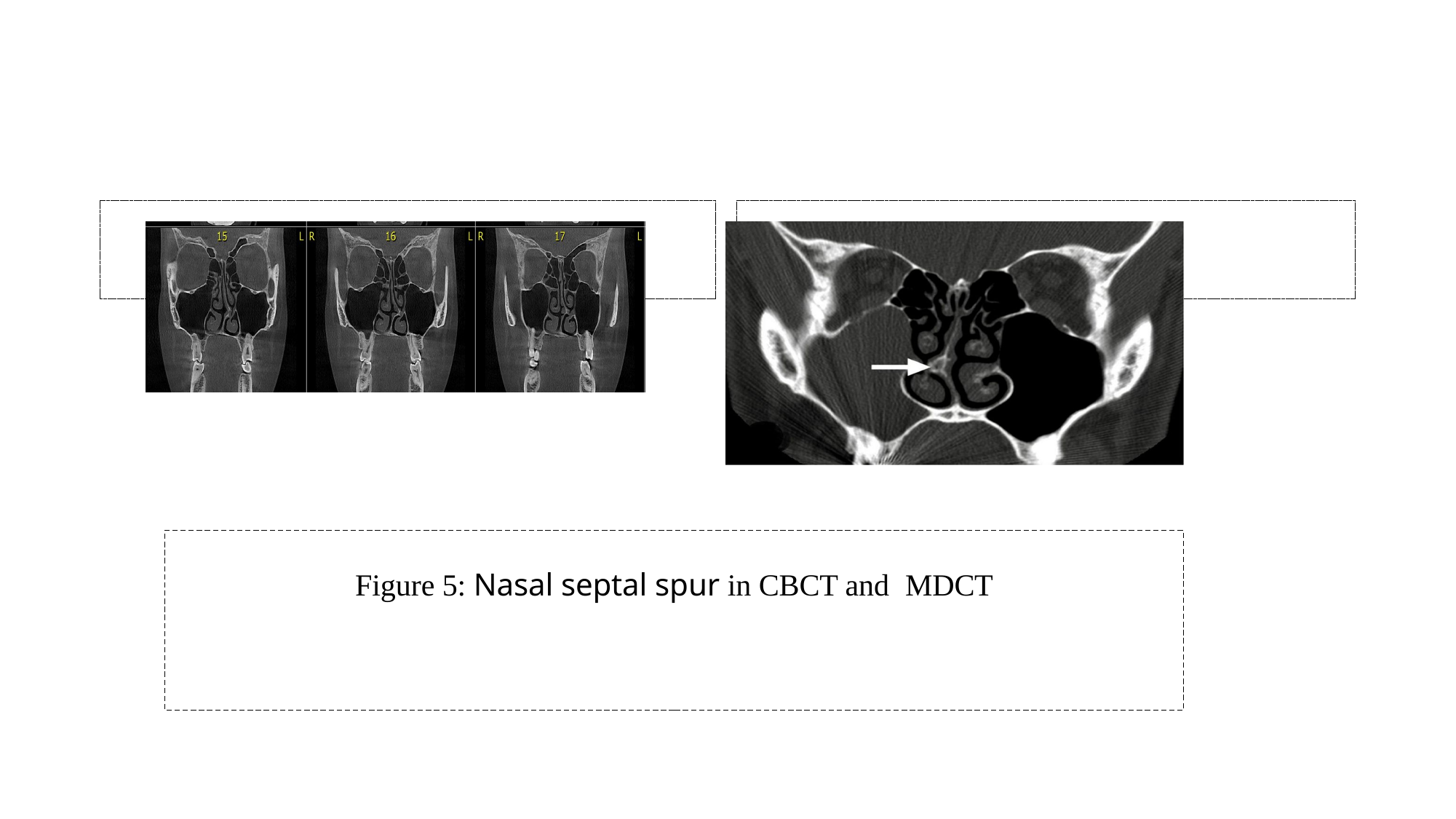

CBCT
CT
# Figure 5: Nasal septal spur in CBCT and 	MDCT
